# Adolescents and young people at the centre: global perspectives and approaches to transform HIV testing, treatment and care

**DOI:** 10.1002/jia2.25581

**Published:** 2020-08-31

**Authors:** Moherndran Archary, Audrey E Pettifor, Elona Toska

**Affiliations:** ^1^ King Edward VIII Hospital Durban South Africa; ^2^ Department of Paediatrics University of KwaZulu‐Natal Durban South Africa; ^3^ Department of Epidemiology Gillings School of Global Public Health University of North Carolina at Chapel Hill Chapel Hill NC USA; ^4^ MRC/Wits Rural Public Health and Health Transitions Research Unit School of Public Health University of the Witwatersrand Witwatersrand South Africa; ^5^ Centre for Social Science Research University of Cape Town Cape Town South Africa; ^6^ Department of Sociology University of Cape Town Cape Town South Africa; ^7^ Department of Social Policy and Intervention University of Oxford Oxford United Kingdom

**Keywords:** HIV, prevention, care, adolescents, young people, approaches

In this special issue of the *Journal of the International AIDS Society,* we focus on research and perspectives that put adolescents and young people at the centre of their HIV prevention and care. Globally over two million adolescents are living with HIV, while many more are at risk of HIV infection [1, 2, 3]. In contrast to reductions globally and in highest burden regions in the general population, estimates suggest that HIV incidence and mortality persist among adolescents and young people. Young people continue be less likely to test for HIV, to link to care in a timely way and to stay engaged in care if they test HIV positive compared to adults [4, 5, 6, 7]. Although, these disparities in access to HIV prevention and care have been documented over the last decade, the evidence base of models, programmes and tools to address the challenges young people face is limited [8, 9, 10, 11]. This special issue documents a series of promising and effective approaches that support adolescents and young people, not only to access and use HIV prevention and care, but also to live whole and fulfilling lives. The articles in this issue bring together evidence and lessons learned from a number of global settings, unified by one clear message: young people need and want to be at the centre of their HIV prevention and care programmes.

Changes in HIV‐related policies and guidelines can have a positive impact in HIV‐related outcomes among adolescents and young people. The move to universal test and treat in 2016 for people living with HIV has had dramatic impacts on the number of people living with HIV who have started antiretroviral therapy (ART) and overall better treatment outcomes in many settings [18]. This is no different for adolescents. Analyses of national data from Thailand by Teeraananchai et al. investigates the impact of shifting from ART initiation based on CD4 values to immediate ART initiation upon HIV‐positive test results [18]. Their analyses of data from over 51,000 15‐ to 24‐year‐old young people show that universal test‐and‐treat can improve linkages to care and reduce mortality among young people, compared to CD4‐driven ART initiation.

Young people and adolescents have long demanded to be centrally involved in developing and implementing HIV research agendas, programmes and policies [12]. Adolescents and young people have different prevention and care needs based on their personal experiences, priorities and aspirations, developmental stages and the context they live in. The approaches in the HIV response need to acknowledge those differences and adapt accordingly [10]. In this issue, Rufurwadzo et al. – young people working with youth‐led organizations and networks at the regional or global level – write about the importance of including adolescents and young people [13]. They give concrete examples of how young people are involved in the HIV response as advocates, educators, researchers and delivering services both within health facilities and through outreach activities. These examples are a start, but need to become the norm in our research and programming for adolescents.

One area in HIV prevention and care that acknowledges adolescents and young people’s voices and needs is their inclusion in the provision of peer support. Mark et al. define peer support for adolescents living with HIV as “a part of adolescent friendly health services as a class of implementation strategies that can support adolescents to access, engage and sustain treatment” [14]. Bernays et al. present data from the Zvandiri peer support programme in Zimbabwe focusing on the experiences of peer supporters themselves. Their findings on research with a cohort of Community Action Treatment Supporters (CATS) highlight several key considerations that need to be addressed as peer support is ramped up as the preferred model for care and support for adolescents living with HIV, especially in high HIV‐burden low‐resource settings [15]. CATS encountered challenges such as the risk of occupational deductive disclosure and managing the emotional labour of providing care as a peer supporter, but these challenges were eased by the support of other peers and adult mentors. In another example from Thailand, Songtaweesin et al. present data from a randomized controlled trial (RCT) showing that young men who have sex with men and young transgender women in Thailand who were provided with youth‐friendly pre‐exposure prophylaxis (PrEP) services had good retention in care at six months (75%). The addition of an app to support adherence did not add to PrEP adherence compared to youth friendly services, underscoring the importance of youth‐friendly service provision [16]. While the App was found to be acceptable to the young people, this small scale RCT may have been underpowered to detect improved outcomes when added to youth‐friendly services. More work is needed on this topic given the often dynamic and unpredictable nature of adolescent sexuality and partnerships.

While adolescent‐friendly and differentiated service provision is important to improve prevention and care outcomes for adolescents, it is essential to ensure that biomedical solutions which are accessible and available to adolescents and young people are developed and distributed. Amstutz et al. present findings from Lesotho demonstrating how HIV self‐testing allows adolescents more flexibility in how, where and when they test for HIV thus improving the uptake of testing coverage, knowledge of HIV status and linkages to care [17]. HIV self‐testing and mHealth solutions (apps or online platforms) could be critical to maintain our progress towards the global 95‐95‐95 targets among adolescents and young people, particularly in the context of the COVID‐19 pandemic and its consequences on an over‐burdened health system. However, the evidence on both is emerging and additional research is needed to understand when such solutions work, but also when they do not work, so that adjusted and tailored versions can be tested and scaled up.

While specific interventions may be critical to the survival of adolescents and young people, their lives, needs and aspirations must be acknowledged holistically and comprehensively. As millions of adolescents and young people remain at risk of HIV infection or are living with HIV, testing, treatment and care services must be provided with a human‐centred and life course approach. Two papers in this issue provide evidence on the complex needs that adolescent HIV programming must take into account. Bernays et al. discuss the complexities of transitioning to second‐line treatment for adolescents and young people living with HIV and the need for a multi‐faceted supported process [19]. Although immediate adherence improvements were reported, these could only be sustained through the development of “*adherence competencies”* in the context of a gradual transition processes supported by both caregivers and clinicians.

Programmes need to continue to aim to provide comprehensive care for the whole young person when they do engage with them for HIV prevention or care. This is particularly relevant in the context of COVID‐19 pandemic when non‐critical engagements with the healthcare system must be limited. Now more than ever it is clear that we need programmes and services that are adaptive to the needs of young people and that meet them where they are with what they need. Toska et al. present data from a survey among young women in South Africa – including adolescent mothers – highlighting the continued importance of young women’s sexual and reproductive health in addition to HIV prevention and care [20]. Even though nearly 95% of the pregnancies during adolescence among study participants were unintended, the use of hormonal contraception and condoms – dual protection – was very low among young women, independent of their HIV status or whether they had already had a child. While these findings are similar to those of studies among adults, it is discouraging that – despite the dialogue on dual protection in the past two decades – there has not been considerable progress in this area. In another example in this issue, Chimbindi et al. report from experiences of scaling up and delivering a layered package of interventions for adolescent girls and young women: the DREAMS initiative, a partnership funded by the United States’ President’s Emergeney Program for AIDS Relief, in a rural remote region of South Africa [21]. They report, much like Bernays and colleagues, that while many excellent programmes and practices exist for adolescent service delivery, there is limited documentation of what works, especially at scale. Additionally, experiences of scaling up a multi‐component package like DREAMS highlights the tensions between local adaptation, adolescent engagement and ownership and maintaining fidelity to the original programmes. Despite the challenges, we must continue to learn how to best deliver combination and multi‐dimensional programmes for youth.

This supplement was being prepared during the COVID‐19 pandemic, though its content does not include data specific to COVID‐19. Public health measure like physical distancing and limiting non‐essential interactions with the healthcare system are raising challenges for access to services deemed non‐essential in many settings, such as HIV testing, PrEP and contraception, as well as for essential services such as access to ART. However, the pandemic also may provide an opportunity to respond to these challenges with some of the adolescent‐friendly and innovative interventions highlighted in this issue, such as the use of mobile health technology to augment adolescent‐ and youth‐friendly services [16], HIV self‐testing kits [17] and differentiated services [15, 21]. The call by Rojo et al. for the development of long‐acting antiretroviral drugs for adolescents can not only potentially improve acceptability and adherence but also limit the frequency of healthcare interactions [22].

## AVENUES FOR FUTURE RESEARCH

In addition to the work presented in this issue, two commentaries highlight several areas for future research and implementation [23, 24], while another calls for stronger implementation science [25]. Laurenzi et al. call for more research evaluating the effects of interventions designed to improve the mental health of adolescents living with HIV (ALHIV) [23]. They highlight four priorities, including to generate more evidence about preventive mental health interventions for ALHIV, to include mental health outcomes in research on psychosocial interventions for ALHIV, to conduct intervention research that is sensitive to differences amongst ALHIV population, while ensuring that adolescents are involved in intervention design and testing. Stangl et al. focus on the needs of young women in the context of implementing partner notification in the context of HIV case indexing [24]. While partner notification is being implemented in some settings, additional research is needed not only to identify effective models but also to ensure potential social harms are identified and mitigated. Both commentaries call for implementation science to assess availability, accessibility, acceptability and quality of programming for adolescent HIV interventions. Vorkoper and colleagues introduce the Adolescent HIV Prevention and Treatment Implementation Science Alliance (AHISA) as a platform for promoting implementation science lessons learned and building capacity to design and conduct implementation science as it relates to adolescent HIV prevention and care in the African region [25]. Such research is critical to understand how evidence‐based programmes can be implemented at scale rigorously, effectively and cost effectively.

For years, adolescent HIV prevention and care practitioners, advocates and researchers have been calling for the urgent need for more evidence‐based interventions to reduce new HIV infections and improve HIV care outcomes for adolescents and young people. Often the disconnect between the research, programme and policy worlds results in proven interventions for adolescents not being implemented, or programmes not being evaluated to identify barriers to scale up. The content of this special issue provides emerging evidence about what works for young populations in different contexts with regard to improving testing, PrEP use and linkage to and adherence to treatment. As highlighted in the youth viewpoint by Rufurwadzo et al., there is a need for healthcare policymakers and implementors to not be overly paternalistic, but to support and trust them to make good decisions through meaningful engagement [13]. Importantly, this supplement’s content highlights that young people are often the best advocates and custodians of their care if they have the tools and the support they need to make safer and healthier choices. As adolescent HIV prevention and care specialists, providers and researchers, we support this positive trajectory and look forward to additional collaborations among policymakers, implementers and donor agencies to strengthen the evidence base and scale up programmes for adolescents and youth.

## COMPETING INTERESTS

The authors have no competing interests.

## AUTHORS’ CONTRIBUTIONS

MA, AEP and ET co‐conceptualized this editorial and co‐wrote it with feedback from the Journal of International AIDS Society editorial team. All authors have read and approved this.
